# A multifunctional switch for label-free CRISPR/Cas12a sensor with self-driven amplification

**DOI:** 10.1016/j.synbio.2025.07.002

**Published:** 2025-07-05

**Authors:** Po Li, Xueying Lei, Xiaoying Niu, Wen Tian, Zhehuang Li, Songcheng Yu, Peng Zhang

**Affiliations:** a Department of Orthopedic and Soft Tissue, The Affiliated Cancer Hospital of Zhengzhou University & Henan Cancer Hospital, Zhengzhou, 450008, China; b College of Public Health, Zhengzhou University, Zhengzhou, 450001, China

**Keywords:** CRISPR/Cas12a, Molecular switch, Label-free, Toehold, MicroRNA

## Abstract

MicroRNA (miRNA) is promising candidate for non-invasive diagnostic biomarker. Conventional CRISPR/Cas12a-based miRNA detection systems are constrained by reliance on reverse transcription, nucleic acid pre-amplification and costly fluorescently labeled reporters which introduce chemical modification complexity and background noise. To address these limitations, we herein developed a multifunctional switch that integrated target recognition, CRISPR/Cas12a system activation, intrinsic fluorescence signaling, and autonomous signal amplification within a single molecular architecture. As a proof of concept, this switch enabled a label-free CRISPR/Cas12a biosensing for miR-21 detection with a detection limit of 4.8 nM and robust performance in accuracy, precision, and selectivity. This proposed label-free CRISPR/Cas12a platform could be applied for real sample and is a promising candidate for point-of-care miRNA detection.

## 1 Introduction

MicroRNAs (miRNAs), a class of non-coding RNAs with 19–25 nucleotides, serve as critical post-transcriptional regulators of gene expression by binding to complementary sequences in target mRNAs [1,2]. Dysregulation of miRNA expression profiles has been mechanistically linked to a spectrum of human pathologies including oncogenesis, cardiovascular dysfunction and neurodegenerative disorders [[3], [4], [5], [6]]. The clinical value of miRNAs is further improved by their tissue-specific expression patterns, resistance to RNase degradation, and remarkable stability in extracellular biofluids [7]. These advantages render miRNAs promising candidates for non-invasive diagnostic biomarkers. For example, miR-21 is a well-documented oncogenic miRNA that promotes tumor proliferation, metastasis and chemoresistance by its upregulation across multiple malignancies [8,9]. Its elevated serum levels have demonstrated significant diagnostic and prognostic value in breast and colorectal carcinomas [[10], [11], [12]]. Thus, there is demand for robust detection of miRNAs.

Conventional miRNA detection relies predominantly on quantitative reverse transcription PCR (qRT-PCR), a technique that is referred as gold standard method for miRNAs detection [[13], [14], [15], [16]]. Although qRT-PCR achieves high sensitivity, its workflow necessitates reverse transcription steps, specialized thermal cycling instrumentation, and labor-intensive primer design, which would cause high costs and prolonged assay times. Furthermore, qRT-PCR also struggles to discriminate mature miRNAs from precursor isoforms and exhibits susceptibility to enzymatic inhibitors present in clinical specimens, which would lead to compromised specificity. These limitations emphasize the urgent need for innovative detection platforms that combine simplicity, precision, and clinical practicality.

Recent advances in CRISPR (Clustered Regularly Interspaced Short Palindromic Repeats)-based diagnostics have introduced new strategies for nucleic acid detection [17]. CRISPR-associated (Cas) proteins, such as Cas13a and Cas12a, enable sequence-specific target recognition coupled with collateral *trans*-cleavage activity to provide detection with high specificity and sensitivity [18,19]. However, existing CRISPR platforms face critical challenges in miRNA detection. CRISPR/Cas13a system suffers from reporter instability due to their reliance on RNA-fluorophore quencher (FQ) reporters [20,21]. In contrast, CRISPR/Cas12a system utilizing DNA-based FQ *trans*-cleavage substrate has gained more attention owing to the reporter stability [22,23]. However, CRISPR/Cas12a-based approaches require reverse transcription and pre-amplification to convert miRNA targets into DNA activators [[24], [25], [26]]. Recently, strategies employing split crRNA or split activators could enable CRISPR/Cas12a system to directly detect miRNA but exhibited lower *trans*-cleavage efficiency that resulted in less sensitivity [[27], [28], [29], [30], [31], [32], [33]]. Besides, issues with background signal interference from imperfect probe quenching and high costs associated with fluorophore labeling further limit the application of CRISPR/Cas12a system for miRNA detection.

**Fig. 1:**
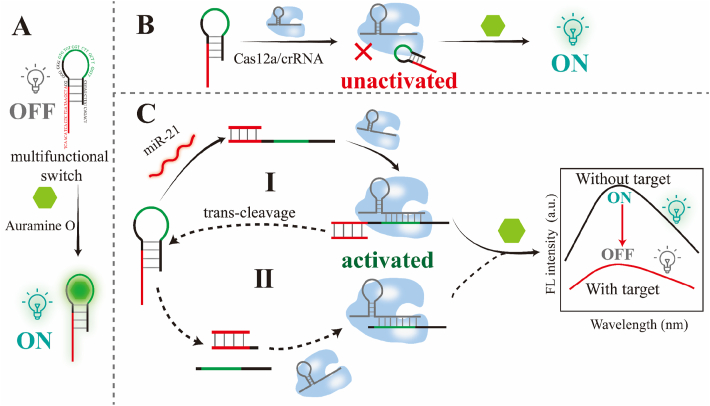
Mechanism illustration of msCRISPR to detect miR-21.

To overcome these challenges, we herein developed a multifunctional switch that synergistically integrated miRNA recognition, CRISPR activation, and signal amplification within a unified and label-free architecture (Fig. 1). The switch contained two functional domains. One domain was a stem structure for miRNA specific sensing via base complementary. The other domain was a multifunctional loop serving as *trans*-cleavage substrate of Cas12a, which contained activator sequence to activate Cas12a and G-quadruplex motif to light up Auramine O. The principle of combination between Auramine O and multifunctional switch could be explained with the aptamer-target recognition, which was initiated by electrostatic attraction and hydrophobic interactions to facilitate initial target proximity. Subsequent molecular docking induced adaptive conformational changes in the tertiary structure of multifunctional switch through an induced-fit process to enable complementary surface topology matching [34]. This structural rearrangement permitted the formation of specific inter-molecular forces including hydrogen bonding, van der Waals contacts, and π-π stacking interactions. Then, Auramine O could be light-up upon its combination with multifunctional switch. [35,36]. As a proof of concept, a label-free CRISPR/Cas12a biosensing for miR-21 detection by application of this multifunctional switch was developed, which was called msCRISPR for short. In the absence of miR-21, the multifunctional switch maintained its structural integrity to sequester the Cas12a activator sequence within its constrained loop domain. This spatial confinement imposed steric hindrance that prevented activator exposure to preclude CRISPR/Cas12a complex activation and consequent *trans*-cleavage activity. Conversely, upon miR-21 hybridization, toehold-initiated strand displacement induced irreversible probe unfolding to liberate the linearized activator sequence from the constrained loop domain. This liberated activator then recruited and activated the Cas12a ribonucleoprotein complex to trigger indiscriminate *trans*-cleavage of nearby single-stranded DNA substrates including the loop domain of intact multifunctional switches, which would decrease the Auramine O fluorescence intensity and release more Cas12a activators. Crucially, the resultant cleavage feedback loop propagated signal amplification through iterative activator release and Cas12a reactivation cycles to establish a self-driven amplification circuit without dependency on exogenous enzymes or amplification techniques. This msCRISPR circumvented reverse transcription, fluorophore labeling and pre-amplification steps in traditional CRISPR/Cas12a detection of miRNA, offering a cost-effective solution for point-of-care testing of miRNA.

## 2 Materials and methods

### 2.1 Reagents and materials

Cas12a protein was purchased from Editgene Co., Ltd. (Guangzhou, China). The 10 × cleavage buffer r2.1 (50 mM NaCl, 10 mM Tris-HCl, 10 mM MgCl_2_, pH 7.9) was used for CRISPR/Cas12a reactions. Oligonucleotides (sequences provided in Table S1) were synthesized and HPLC-purified by Sangon Biotech (Shanghai, China). All nucleic acid solutions were prepared using DEPC-treated water (for RNA) or TE buffer (for DNA). Auramine O fluorescent dye was obtained from Aladdin Biochemical Technology Co., Ltd. (Shanghai, China), with its fluorescence reaction buffer containing 20 mM Tris-HCl, 20 mM KCl, and 25 mM MgCl_2_ (pH 7.4).

### 2.2 Feasibility analysis

**Auramine O-Switch Fluorescence Characterization**. Switch solutions (0–20 μM) were prepared by serial dilution. For each reaction, 1 μL switch solution, 6 μL Auramine O (20 μM) and 13 μL fluorescence reaction buffer was mixed and incubated at room temperature (25 °C) for 30 min incubation. Fluorescence measurement was performed using an FS5 fluorescence spectrophotometer from Edinburgh Instruments (Livingston, UK) with excitation at 480 nm and emission of 510–600 nm.

**Switch Inhibition of Cas12a Activity**. The Cas12a/crRNA1 complex was pre-formed by incubating 0.5 μL Cas12a (1 μM), 0.5 μL crRNA2 (1 μM), and 2 μL buffer r2.1 for 10 min at 25 °C. This complex was then mixed with 10 μL Milli-Q water, 1 μL FAM-BHQ reporter probe (10 μM), and 1 μL switch (20 μM) in a total reaction volume of 20 μL. After 30 min incubation at 25 °C, fluorescence was measured using identical instrument parameters as above with excitation at 493 nm.

**Switch as Cas12a Trans-cleavage Substrate**. The Cas12a/crRNA2 complex was prepared by incubating 0.5 μL Cas12a (1 μM), 0.5 μL crRNA1 (1 μM), and 2 μL buffer r2.1 for 10 min at 25 °C. Subsequently, 3 μL complex was mixed with 9 μL fluorescence buffer, 1 μL switch (20 μM), 0.5 μL target ssDNA2 (1 μM), and 6.5 μL Auramine O solution (20 μM). Following 30 min incubation at 25 °C, fluorescence was measured at 548 nm with 480 nm excitation.

**miR-21 Sensing Validation**. For direct detection, 1 μL miR-21 (20 μM) was incubated with 1 μL switch (20 μM) and 8 μL fluorescence buffer for 30 min at 25 °C. Mixtures were heat-inactivated (95 °C, 20 min) and analyzed by 15 % native PAGE and fluorescence measurement after adding 6 μL Auramine O (100 μM) and 4 μL fluorescence buffer.

**CRISPR/Cas12a Detection System**. The optimized detection system contained 1 μL miR-21 (20 μM), 1 μL switch (20 μM), 8 μL fluorescence buffer, 3 μL Cas12a/crRNA1 complex (pre-formed as above), and 7 μL reaction buffer. After 30 min incubation at 25 °C, reactions were heat-inactivated (95 °C, 20 min) and analyzed by PAGE and fluorescence as described.

### 2.3 Condition optimization

**Switch Concentration Screening**. Reactions containing 1 μL switch (250 nM-1.5 μM), 1 μL miR-21 (1 μM), 9 μL buffer, 3 μL Cas12a complex, and 6 μL Auramine O (30 μM) were incubated at 25 °C for 30 min. After post-inactivation (95 °C, 20 min), fluorescence was measured at 548 nm.

**Auramine O Concentration Optimization**. Using fixed switch (1 μM) and miR-21 (10 μM), Auramine O concentrations (10–40 μM) were tested following the protocol in switch concentration screening.

**Concentration of Cas12/crRNA**. With optimized switch (1 μM) and Auramine O (30 μM), Cas12a/crRNA1 concentrations (5–35 nM) were evaluated using 1 μL miR-21 (10 μM) in standard reaction conditions. The CRISPR/Cas12a reaction system was prepared by mixing Cas12a and crRNA1 at a 1:1 ratio to achieve the desired concentrations, followed by the addition of 2 μL buffer r2.1.

**Thermal Parameter Optimization**. Temperature (4–45 °C) and time (10–40 min) parameters were systematically tested under established optimal conditions (1 μM switch, 30 μM Auramine O, 25 nM Cas12a/crRNA1).

### 2.4 Detection procedures of msCRISPR

1 μL miR-21 sample was mixed with 1 μL of switch (20 μM) and 8 μL of fluorescence reaction buffer (20 mM Tris-HCl, 20 mM KCl, 25 mM MgCl_2_, pH 7.4) in a 0.2 mL microcentrifuge tube. The mixture was incubated at 25 °C for 30 min to facilitate hybridization between miR-21 and switch. Subsequently, 3 μL of pre-assembled Cas12a/crRNA1 complex (incubation of 0.5 μL Cas12a, 0.5 μL 1 μM crRNA1, and 2 μL 10 × cleavage buffer r2.1 for 10 min at 25 °C) and 7 μL of fluorescence reaction buffer containing Auramine O was added to achieve a final volume of 20 μL. The complete reaction system was incubated at 25 °C for 30 min to allow Cas12a-mediated *trans*-cleavage of the unbound switch. The mixture was heat-inactivated at 95 °C for 20 min to terminate the reaction. Fluorescence intensity was measured using an FS5 fluorescence spectrophotometer with excitation at 480 nm and emission at 548 nm. All experiments included negative controls lacking miR-21 to establish baseline signals.

### 2.5 Real sample detection

In order to evaluate the implementation of the suggested technique in real sample, osteosarcoma tissue and normal cartilaginous tissue samples were obtained from two patients diagnosed with osteosarcoma. The total RNA from tissues was extracted using A G RNAex Pro Reagent, which was obtained from Accurate Biotechnology Co., Ltd. (Hunan, China). The miR-21 levels of real samples were determined according to aforementioned procedures.

## 3 Results and discussion

### 3.1 Feasibility of the assay

**Fig. 2:**
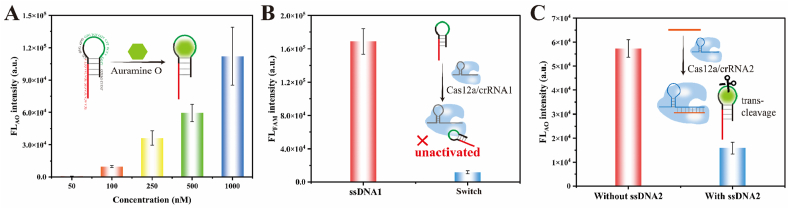
The properties of switch. (A) The fluorescence response of switch to Auramine O fluorescent dye, concentration of which was fixed at 20 μM. FL_AO_ intensity was the fluorescence intensity of Auramine O, and the abscissa was the different concentration of switch. (B) The FAM fluorescence of switch inhibitory effects of Cas12a activity. FL_FAM_ intensity was the fluorescence intensity of FAM, and the abscissa was the different activators. (C) The fluorescence of switch as Cas12a *trans*-cleavage substrate with or without ssDNA2. The inset was a schematic of the reaction of the sections. (Error bars: SD, n = 3).

**Auramine O-Switch Fluorescence Characterization**. To confirm the binding interaction between the switch and Auramine O fluorescent dye, varying concentrations of switch were incubated with Auramine O at 25 °C for 30 min, followed by measurement of relative fluorescence intensity. As shown in Fig. 2A, a concentration-dependent increase in fluorescence intensity was observed. Higher switch concentration resulted in stronger fluorescence signal. This trend clearly demonstrated the interaction between switch and Auramine O to light-up fluorescence.

**Switch Inhibition of Cas12a Activity**. To confirm the inhibitory effect of switch on Cas12a activity, we used it as an activator in the CRISPR/Cas12a reaction system and compared its fluorescence with that of the classical activator ssDNA1. As shown in Fig. 2B, ssDNA1 activated the *trans*-cleavage activity of Cas12a to generate a fluorescence intensity over fivefold higher than that of switch. Moreover, fluorescence for switch was almost equal to that of the blank control, which indicated its negligible binding ability to the Cas12a/crRNA1 complex. Thus, intact switch could not activate CRISPR/Cas12a system to generate fluorescence.

**Switch as Cas12a Trans-cleavage Substrate**. To validate the capability of switch as a *trans*-cleavage substrate for Cas12a, we used another ssDNA with different sequence as activator to trigger the *trans*-cleavage activity of Cas12a. Switch was used as the *trans*-cleavage substrate. Auramine O was added for fluorescence measurement. As shown in Fig. 2C, the fluorescence intensity for the blank control was about 4 times higher than that in the presence of the activator ssDNA2. It was demonstrated that the switch could act as a *trans*-cleavage substrate for Cas12a.

**Fig. 3:**
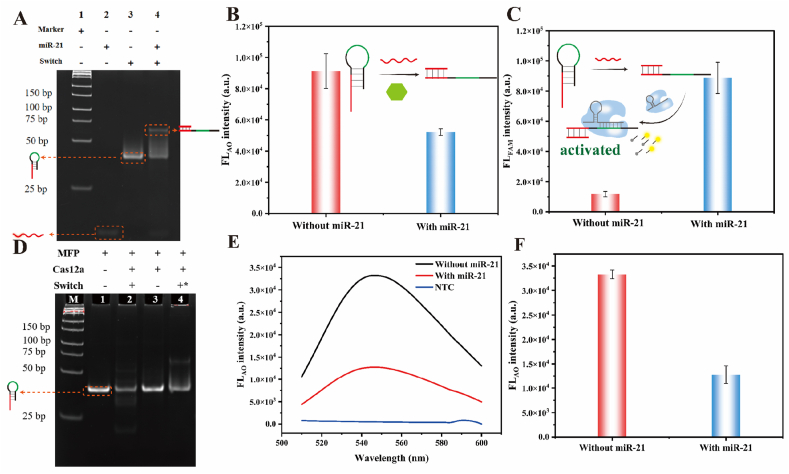
miR-21 detection. (A) Polyacrylamide gel electrophoresis analysis of miR-21 and switch. The final concentration of all strands was 1 μM. (B) The fluorescence intensity of Auramine O of miR-21 and switch. (C) The fluorescence intensity of FAM of miR-21, switch and CRISPR/Cas12a system. (D) Polyacrylamide gel electrophoresis analysis of miR-21, switch and Cas12a/crRNA1. The final concentration of all strands was 1 μM. (E–F) The Auramine O fluorescence intensity of miR-21, switch and Cas12a/crRNA1. The final concentration of all strands was 1 μM. The “+” represented the presence and the “-” represented the absence of the corresponding component. (Error bars: SD, n = 3).

**miR-21 Sensing Validation**. To confirm whether miR-21 could open the switch, miR-21 was pre-incubated with switch and the mixture was subsequently analyzed using polyacrylamide gel electrophoresis and fluorescence measurement. The electrophoresis results were presented in Fig. 3A. Compared with miR-21 in lane 2 and switch in lane 3, the mixture had a new band in lane 4, which indicated that switch could be successful opened by miR-21. In addition to electrophoretic analysis, the interaction between miR-21 and switch was further investigated by monitoring fluorescence changes of Auramine O at 548 nm. The loop region of the intact switch could serve as an aptamer to light-up Auramine O. Therefore, in the presence of miR-21, switch was opened to disrupt its specific binding to Auramine O, which resulted in decreased fluorescence intensity (Fig. 3B). These results demonstrated the effectiveness of switch in detecting target miR-21.

Besides, to confirm whether the opened switch trigger the CRISPR/Cas12a system, the FAM fluorescence measurement was conducted, which results was shown as Fig. 3C. In the presence of miR-21, the opened switch could activate the *trans*-cleavage of Cas12a, resulting in the increasing FAM fluorescence. This determined that the opened switch could trigger the *trans*-cleavage of Cas12a.

**CRISPR/Cas12a Detection System**. To investigate whether the reaction products of miR-21 and the switch could activate CRISPR/Cas12a to trigger the *trans*-cleavage of the switch, the products were analyzed using polyacrylamide gel electrophoresis as well as fluorescent measurement. As illustrated in Fig. 3D, the switch residue obviously decreased in the present of miR-21 (lane 2) when compared with the control (lane 3), which was not observed in the group of deactivated Cas12a (lane 4). It was demonstrated that miR-21 could effectively open the switch to initiate the *trans*-cleavage of the switch. In addition, fluorescence monitoring was also applied to evidence the feasibility of miR-21 detection with switch-mediated CRISPR/Cas12a system. The results were displayed in Fig. 3E–F. In the presence of miR-21, the switch underwent structural opening to expose the activator sequence to activate CRISPR/Cas12a that subsequently *trans*-cleaved the switch to cause a measurable decrease in Auramine O fluorescence intensity (Fig. 3E–F). These findings demonstrated that the switch function dually as a recognition element for miR-21 detection and as a reporter probe for fluorescent signal transduction.

### 3.2 Optimization of experimental conditions

**Fig. 4:**
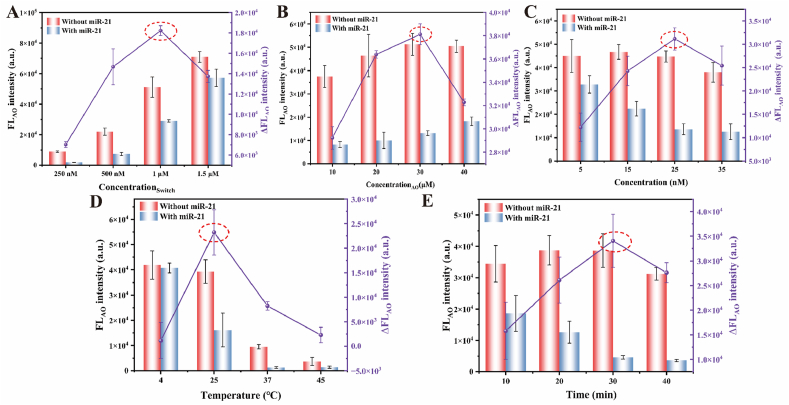
The optimization results of experimental conditions. The results of (A) switch concentration, (B) Auramine O concentration, (C) Cas12a/crRNA1 concentration, (D) temperature and (E) time for CRISPR/Cas12a reaction. ΔFL_AO_ intensity was the difference between with and without miR-21. (Error bars: SD, n = 3).

**Switch Concentration**. The switch governed both miR-21 recognition and fluorescence signal generation, the concentration of which was crucial to improve the performance of the miR-21 detection. As demonstrated in Fig. 4A, a concentration-dependent increase in fluorescence intensity was observed for both in the present of miR-21 and the control groups. The maximal intergroup signal differential occurred at 1 μM switch, which was selected as the optimal concentration to balance target sensing capacity and background signal suppression.

**Auramine O Concentration**. The fluorescence output dependency on Auramine O concentration was subsequently investigated (Fig. 4B). Fluorescence intensity exhibited a proportional relationship with dye concentration across all tested conditions. The maximal fluorescence differential was achieved at 30 μM, which was optimal for minimizing background interference while maintaining detection sensitivity.

**Cas12a/crRNA1 Concentration**. The concentration of Cas12a/crRNA1 complex was a critical determinant of *trans*-cleavage activity. The results of the optimization through concentration-gradient experiments were shown in Fig. 4C. Fluorescence intensity in the control group remained stable across tested concentrations (5–35 nM), whereas the miR-21 group exhibited progressive signal attenuation with increasing complex concentration. The maximal signal differentiation was observed at 25 nM, which was optimal for balancing enzymatic activity and reagent economy.

**Temperature and Time for CRISPR/Cas12a Reaction**. The CRISPR/Cas12a reaction temperature was optimized to maximize enzymatic activity while preserving intact switch structure (Fig. 4D). At 4 °C, high fluorescence signal was detected for both groups with or without miR-21. Little intergroup differentiation of fluorescence intensity indicated neglect Cas12a activation. While temperatures elevated, especially at 37 °C and 45 °C, the fluorescence signal reduced dramatically for the control group. It was revealed that switch suffered from thermal destabilization at high temperature. In the present of miR-21, the fluorescence signal decreased greater degree than that of control group due to both the thermolability of switch and the increased activity of Cas12a. The optimal balance between enzymatic activity and probe stability was achieved at 25 °C to yield maximal signal differentiation. Reaction time optimization revealed distinct kinetic profiles between groups (Fig. 4E). In the present of miR-21, there was a time-dependent signal decrease with a plateau from 30 min to 40 min. In contrast, the fluorescence of control group remained stable up to 30 min. The maximal intergroup contrast at 30 min established this duration as optimal for *trans*-cleavage of switch by CRISPR/Cas12a system.

### 3.3 Analytical performance

**Fig. 5:**
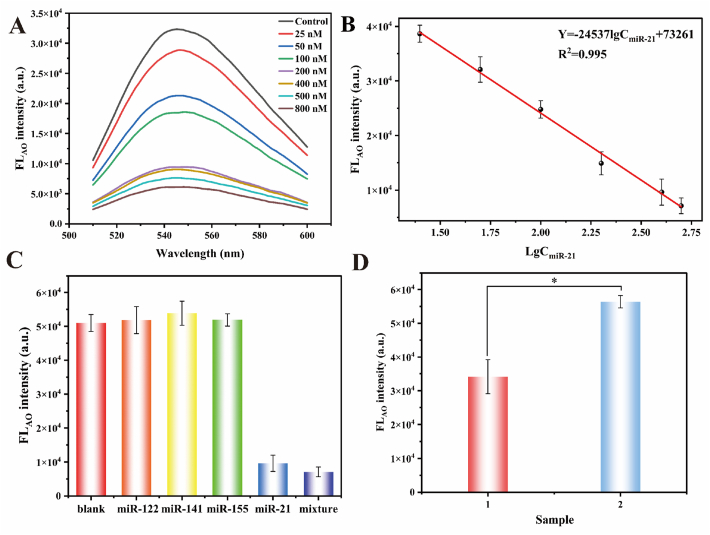
Analytical performance and real sample of miR-21 detection. (A) Fluorescence intensity corresponding to different miR-21 concentrations. (B) Calibration curve of the fluorescence as a function of the logarithm of miR-21 concentration from 25 nM to 500 nM. (C) The specificity of miR-21. Concentrations of interferences (miR-122, miR-144 and miR-155): 100 nM, concentration of miR-21: 50 nM. (D) Real sample detection in the osteosarcoma tissue and normal cartilaginous samples from two osteosarcoma patients. Group 1 was the osteosarcoma tissue and group 2 was the normal cartilaginous tissue. ∗ represented *P* < 0.5. (Error bars: SD, n = 3).

**Standard Curve and Linear Range**. Under the optimized experimental conditions, a series of miR-21 standard solutions with concentration gradients were analyzed according to the detection procedures. The standard curve was constructed by plotting the Auramine O fluorescence intensity (Y) on the Y-axis against the logarithm of miR-21 concentration (lgC_miR-21_, where C_miR-21_ was expressed in nM) on the X-axis (Fig. 5A–B). Within the concentration range of 25 nM–500 nM, a significant linear relationship was observed between the fluorescence signal and the logarithm of miR-21 concentration. The linear regression equation was determined as: Y = −24,573 × lgC_miR-21_+73261. This linear correlation demonstrated the ability of proposed method to quantitatively detect miR-21 across a wide dynamic range.

**Limit of Detection (LOD)**. LOD was established through statistical analysis of six independent blank measurements. The threshold fluorescence signal was defined as the mean intensity of blank samples plus three times their standard deviation. This threshold value was subsequently interpolated into the linear regression equation of standard curve (Y = −24,573 × lgC_miR-21_+74261), yielding an LOD of 4.8 nM for miR-21.

**Specificity**. To assess the selectivity of the proposed method, the fluorescence responses of three non-target miRNAs were measured and compared with that of the target miR-21. As illustrated in Fig. 5C, the fluorescence intensity observed for miR-21, as well as for a mixture containing miR-21 and other miRNAs, was significantly lower than that of the blank control and the individual non-target miRNAs. This distinct signal differentiation demonstrated the high selectivity of the method toward miR-21.

**Recovery and Precision**. To assess the accuracy and precision of the developed method, spiked samples with miR-21 at three concentration levels (low: 25 nM, medium: 100 nM, and high: 500 nM) were prepared and detected according the detection procedures. The recovery rates and relative standard deviations (*RSD*s) were calculated. As presented in Table S2, the recovery rates of miR-21 spiked samples fluctuated between 99.72 % and 102.34 %. And the *RSD* values were within the range from 4.03 % to 9.57 %. These results demonstrated that the method developed in this study possesses good accuracy and precision.

### 3.4 Real sample detection

In order to the possible applicability of the proposed sensor for the detection of miR-21 in real samples, an analysis was conducted on the osteosarcoma tissue and normal cartilaginous tissue samples from two osteosarcoma patients. As shown in Fig. 5D, the observed miR-21 levels in the osteosarcoma tissue were markedly elevated compared to those in the normal cartilaginous tissue (*P* < 0.5). Taken together, these findings suggest that the proposed method has the potential for therapeutic applications.

## 4 Conclusion

Conventional CRISPR/Cas12a-based miRNA detection systems face limitations including the requirement for reverse transcription and pre-amplification steps to convert miRNA targets into DNA activators. Besides, canonical fluorescent reporters suffer from high background signals due to imperfect probe quenching, complicated chemical modification and high cost of fluorophore labeling. To address these issues, we developed a molecular switch with multifunction: (1) direct miRNA target recognition, (2) intrinsic light-up Auramine O fluorescence, (3) CRISPR/Cas12a system activation after target miRNA recognition, and (4) self-driven signal amplification via Cas12a-mediated *trans*-cleavage. Leveraging this integrated probe, we established a label-free CRISPR/Cas12a biosensing platform for direct miR-21 detection. The operational mechanism involved miR-21-induced structural reorganization of the switch to expose a loop-embedded activator sequence that triggers Cas12a activation. Activated Cas12a subsequently executed *trans*-cleavage of the loop domain of switch, which would induce dual effects: immediate fluorescence attenuation due to Auramine O dissociation and release of additional activator sequences that propagate cascading signal amplification. This self-sustaining cycle enabled miR-21 detection with a calculated limit of detection of 4.8 nM. The proposed label-free CRISPR/Cas12a sensor demonstrated superior analytical performance, including no significant cross-reactivity to other miRNAs, recovery from 99.72 % to 102.34 % and good precision. The key advantages of msCRISPR over conventional methods, including direct miRNA detection capability, cost-effective label-free design, enhanced signal fidelity, and autonomous signal amplification, collectively establish it as a highly promising platform for point-of-care diagnostics. These breakthroughs could enable the development of portable, user-friendly devices for rapid miRNA profiling in resource-limited settings to empower early disease screening at the community level. Furthermore, by eliminating complex instrumentation and specialized operators, this technology may accelerate the clinical translation of miRNA biomarkers into routine precision medicine practice.

## CRediT authorship contribution statement

**Po Li:** Writing – review & editing, Supervision. **Xueying Lei:** Writing – review & editing, Visualization, Methodology, Investigation, Data curation. **Xiaoying Niu:** Writing – review & editing, Supervision. **Wen Tian:** Writing – review & editing. **Zhehuang Li:** Writing – review & editing. **Songcheng Yu:** Writing – review & editing, Writing – original draft, Supervision, Funding acquisition, Conceptualization. **Peng Zhang:** Writing – review & editing, Funding acquisition.

## Declaration of competing interest

The authors declare that they have no known competing financial interests or personal relationships that could have appeared to influence the work reported in this paper.
