# Supplementary material for: A multifunctional switch for label-free CRISPR/Cas12a sensor with self-driven amplification
Source: Synth Syst Biotechnol. 2025 Jul 5;10(4):1208–14. doi: 10.1016/j.synbio.2025.07.002 (PMC12304900; doi:10.1016/j.synbio.2025.07.002)
Supplement: Multimedia component 1 [file mmc1.docx]

**A multifunctional switch for label-free CRISPR/Cas12a sensor with self-driven amplification**

Po Li^a^, Xueying Lei^b^, Xiaoying Niu^a^, Wen Tian^a^, Zhehuang Li^a^, Songcheng Yu^b^ *, Peng Zhang^a^ *

^a^Department of Orthopedic and Soft Tissue, The Affiliated Cancer Hospital of Zhengzhou University & Henan Cancer Hospital, Zhengzhou 450008, China

^b^College of Public Health, Zhengzhou University, Zhengzhou 450001, China

*Corresponding Author: Peng Zhang, zdzp@zzu.edu.cn; Songcheng Yu, scyu@zzu.edu.cn

**1. Sequences of nucleic acids**

**Table S1.** The oligonucleotide sequences of RNA and DNA

| Oligonucleotide | Sequence (5’-3’) |
| --- | --- |
| multifunctional switch | TCAACATCAGTCTGATAAGCTACGGGGGAGGGTGTGTGGTCTTGCTTGGTTCGTAGCTTATCAGACT |
| crRNA1 | UAAUUUCUACUAAGUGUAGAUAACCAAGCAAGACCACACAC |
| miR-21 | UAGCUUAUCAGACUGAUGUUGA |
| miR-141 | UAACACUGUCUGGUAAAGAUGG |
| miR-122 | UGGAGUGUGACAAUGGUGUUUG |
| miR-155 | UUAAUGCUAAUCGUGAUAGGGGU |
| ssDNA1 | GGTGTGTGGTCTTGCTTGGTT |
| reporter | 6-FAM-TTTTTT-BHQ-1 |
| crRNA2 | UAAUUUCUACUAAGUGUAGAUCCGCCGAACGCACGCGAUCC |
| ssDNA2 | GGATCGCGTGCGTTCGGCGG |

**2. Recovery and *RSD* of proposed assay in real samples**

**Table S2.** The proposed sensor for miR-21 determination in real samples (n = 3)

| Added (nM) | Found (nM) | Recovery (%) | *RSD* (%) |
| --- | --- | --- | --- |
| 25 | 511.68±4.26 | 102.34% | 4.03% |
| 100 | 95.27±4.24 | 95.27% | 6.44% |
| 500 | 498.62±4.23 | 99.72% | 9.57% |
